# Poly[(μ_4_-pyridine-2,3-dicarboxyl­ato)lead(II)]

**DOI:** 10.1107/S1600536811000419

**Published:** 2011-01-15

**Authors:** Shie Fu Lush, Fwu Ming Shen

**Affiliations:** a Department of General Education Center, Yuanpei University, HsinChu 30015, Taiwan; b Department of Biotechnology, Yuanpei University, HsinChu 30015, Taiwan

## Abstract

In the title coordination polymer, [Pb(C_7_H_3_NO_4_)]_*n*_, the Pb^II^ ion is eight-coordinated in a distorted square-anti­prismatic geometry formed by one pyridine N atom and seven carboxyl­ate O atoms from four pyridine-2,3-dicarboxyl­ate (pda) anions. In the pda anion, the dihedral angles between the pyridine ring and carboxyl­ate groups are 19.5 (6) and 73.3 (6)°. The carboxyl­ate groups of the pda anions bridge the Pb ions, forming a two-dimensional coordination polymer parallel to (100). Weak inter­molecular C—H⋯O hydrogen boning is present in the crystal structure.

## Related literature

For the coordination modes of the pyridine-2,3-dicarboxyl­ate anion, see: Aghabozorg *et al.* (2007 ▶); Baruah *et al.* (2007 ▶); Li *et al.* (2006 ▶). For the biological activity of pyridine-2,3-dicarb­oxy­lic acid, see: Xiang *et al.* (2006 ▶); Yang *et al.* (2006 ▶); Zhang *et al.* (2008 ▶). For the inert lone-pair effect, see: Liat *et al.* (1998 ▶). For longer Pb—O bonds, see: Mao *et al.* (2006 ▶); Yang *et al.* (2010 ▶).
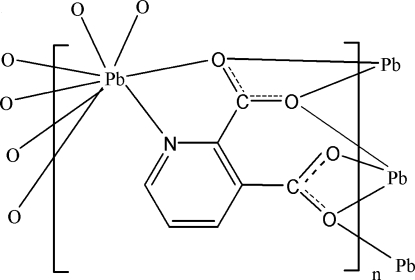


## Experimental

### 

#### Crystal data

[Pb(C_7_H_3_NO_4_)]
*M* *_r_* = 372.30
Monoclinic, 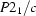

*a* = 11.6943 (9) Å
*b* = 4.5392 (4) Å
*c* = 14.1636 (12) Å
β = 90.046 (2)°
*V* = 751.84 (11) Å^3^
*Z* = 4
Mo *K*α radiation
μ = 22.42 mm^−1^
*T* = 297 K
0.54 × 0.23 × 0.04 mm

#### Data collection

Bruker SMART CCD area-detector diffractometer
Absorption correction: multi-scan (*SADABS*; Bruker, 2001 ▶) *T*_min_ = 0.659, *T*_max_ = 1.000
4013 measured reflections
1484 independent reflections
1336 reflections with *I* > 2σ(*I*)
*R*_int_ = 0.125

#### Refinement

*R*[*F* ^2^ > 2σ(*F* ^2^)] = 0.073
*wR*(*F* ^2^) = 0.204
*S* = 1.13
1484 reflections
118 parameters
H-atom parameters constrained
Δρ_max_ = 4.56 e Å^−3^
Δρ_min_ = −5.06 e Å^−3^

### 

Data collection: *SMART* (Bruker, 2000 ▶); cell refinement: *SAINT* (Bruker, 1999 ▶); data reduction: *SAINT*; program(s) used to solve structure: *SHELXTL* (Sheldrick, 2008 ▶); program(s) used to refine structure: *SHELXTL*; molecular graphics: *PLATON* (Spek, 2009 ▶); software used to prepare material for publication: *PLATON*.

## Supplementary Material

Crystal structure: contains datablocks global, I. DOI: 10.1107/S1600536811000419/xu5124sup1.cif

Structure factors: contains datablocks I. DOI: 10.1107/S1600536811000419/xu5124Isup2.hkl

Additional supplementary materials:  crystallographic information; 3D view; checkCIF report

## Figures and Tables

**Table 1:** Selected bond lengths (Å)

Pb—N	2.651 (7)
Pb—O1^i^	2.816 (7)
Pb—O1^ii^	2.911 (6)
Pb—O2	2.592 (7)
Pb—O2^i^	2.566 (9)
Pb—O3^iii^	2.397 (9)
Pb—O3^ii^	2.754 (9)
Pb—O4^ii^	2.845 (7)

Symmetry codes: (i) 
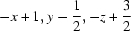
; (ii) 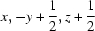; (iii) 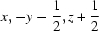.

**Table 2:** Hydrogen-bond geometry (Å, °)

*D*—H⋯*A*	*D*—H	H⋯*A*	*D*⋯*A*	*D*—H⋯*A*
C5—H5*A*⋯O3^iii^	0.93	2.57	3.164 (13)	122

Symmetry code: (iii) 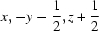.

**Fig. 1.**
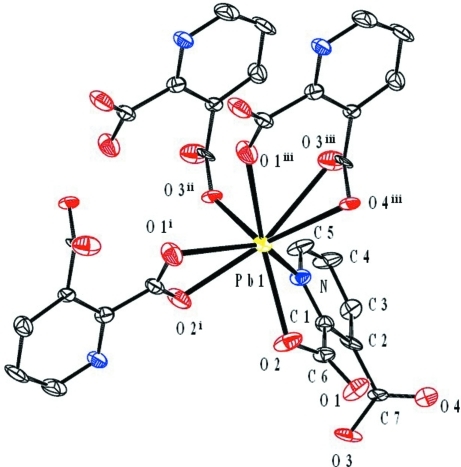
View of the title compound with the atom numbering scheme. Displacement ellipsoids for non-H atoms are drawn at the 50% probability level. H atoms have been omitted for clarity.[Symmetry codes: (i) -x + 1, y - 1/2, -z + 3/2; (ii) x, -y - 1/2, z + 1/2; (iii) x, -y + 1/2].

## supplementary crystallographic information

### Comment

The pyridine-2,3-dicarboxylic acid (pdaH_2_) is a typical chealated-form ligand. Its biological importance has been described in several literatures (Xiang *et al.*, 2006; Yang *et al.*, 2006; Zhang *et al.*, 2008). Pda shows diverse coordination modes, such as monodentate (Baruah *et al.*, 2007), µ_2_-bridging (Aghabozorg *et al.*, 2007), µ_3_-bridging (Li *et al.*, 2006). Here we describe the title compound in which the pda is a µ_4_-bridging ligand (Fig. 1).

The structure of a coordination polymer [Pb(C_7_H_3_NO_4_)]_n_, the lead ion is eight-coordinated with a distorted square-antiprismatic geometry formed by one O-monodentate pda^-2^ ligand, one N,O-bidentate pda^-2^ ligand, one O,O'-bidentate pda^-2^ ligand and one O,O',O''-tridentate pda^-2^ ligand (Table 1). According to "inert-pair effect", the coordination number of Pb^II^ is variable, and the lengths of bonds to Pb^II^ vary in a wide range (Liat *et al.*, 1998). Longer distance is observed between Pb and O1 (2.911 (6) Å); some long Pb—O weak bonds have also been reported in reported lead complexes (Mao *et al.*, 2006; Yang *et al.*, 2010). The carboxylate group of pda^-2^ligand bridges four Pb^II^ ion forming a 2-D framework is constructed.

There are no classic intermolecular hydrogen-bonding in the title compound, but intermolecular C—H···O weak interaction (Table 2) and ring···metal interaction help to stabilize the crystal structure, the *Cg*3 (Pb/O1—O2—C6)···Pb interaction is 3.877 Å (symmetry code: 1 - *x*,-1/2 + *y*,3/2 - *z*).

### Experimental

An aqueous solution (5 ml) containing Pb(NO_3_)_2_ (0.164 g, 0.50 mmol) and 1,2-bis(4-pyridyl)ethane (0.0934 g, 0.50 mmol) was added to an aqueous solution (5 ml) of pyridine-2,3-dicarboxylic acid (0.0838 g, 0.50 mmol), and the mixture was stirred for 30 minutes and then filtered. The solution was placed in a 23 ml Teflon-lined reactor, heated at 423 K for 3 days, then cooled slowly to room temperature. The colorless transparent single crystals of the title compound were obtained in 45.67% yield (based on Pb).

### Refinement

H atoms were positioned geometrically with C—H = 0.93 (aromatic), and were refined using a riding model with *U*_iso_(H) = 1.2*U*_eq_(C).

### Crystal data

**Table:**

[Pb(C_7_H_3_NO_4_)]	*F*(000) = 664
*M**_r_* = 372.30	*D*_x_ = 3.289 Mg m^−^^3^
Monoclinic, *P*2_1_/*c*	Mo *K*α radiation, λ = 0.71073 Å
Hall symbol: -P 2ybc	Cell parameters from 2856 reflections
*a* = 11.6943 (9) Å	θ = 2.3–26.0°
*b* = 4.5392 (4) Å	µ = 22.42 mm^−^^1^
*c* = 14.1636 (12) Å	*T* = 297 K
β = 90.046 (2)°	Parallelepiped, colorless
*V* = 751.84 (11) Å^3^	0.54 × 0.23 × 0.04 mm
*Z* = 4	

### Data collection

**Table:**

Bruker SMART CCD area-detector diffractometer	1484 independent reflections
Radiation source: fine-focus sealed tube	1336 reflections with *I* > 2σ(*I*)
graphite	*R*_int_ = 0.125
Detector resolution: 9 pixels mm^-1^	θ_max_ = 26.0°, θ_min_ = 1.7°
ω scan	*h* = −14→10
Absorption correction: multi-scan (*SADABS*; Bruker, 2001)	*k* = −5→5
*T*_min_ = 0.659, *T*_max_ = 1.000	*l* = −17→17
4013 measured reflections	

### Refinement

**Table:**

Refinement on *F*^2^	Primary atom site location: structure-invariant direct methods
Least-squares matrix: full	Secondary atom site location: difference Fourier map
*R*[*F*^2^ > 2σ(*F*^2^)] = 0.073	Hydrogen site location: inferred from neighbouring sites
*wR*(*F*^2^) = 0.204	H-atom parameters constrained
*S* = 1.13	*w* = 1/[σ^2^(*F*_o_^2^) + (0.1477*P*)^2^] where *P* = (*F*_o_^2^ + 2*F*_c_^2^)/3
1484 reflections	(Δ/σ)_max_ = 0.001
118 parameters	Δρ_max_ = 4.56 e Å^−^^3^
0 restraints	Δρ_min_ = −5.06 e Å^−^^3^

### Special details

**Table:**

Experimental. Elemental analysis: calculated for C_7_H_3_NO_4_Pb: (Mr=372.29) C, 22.56; H, 0.81; N, 3.76%. Found:*C*,22.47; H, 0.89; N, 3.85%. IR data (cm^-1^): 3429(*s*), 1602(*s*), 1579(*s*), 1551(*s*), 1459(w), 1385(*s*), 1276(w), 1236(w), 1105(*m*), 871(*m*), 825(*m*), 779(*m*), 711(*s*), 700(*m*), 660(*m*), 603(w), 534(w), 443(w).
Geometry. Bond distances, angles *etc*. have been calculated using the rounded fractional coordinates. All su's are estimated from the variances of the (full) variance-covariance matrix. The cell e.s.d.'s are taken into account in the estimation of distances, angles and torsion angles
Refinement. Refinement on *F*^2^ for ALL reflections except those flagged by the user for potential systematic errors. Weighted *R*-factors *wR* and all goodnesses of fit *S* are based on *F*^2^, conventional *R*-factors *R* are based on *F*, with *F* set to zero for negative *F*^2^. The observed criterion of *F*^2^ > σ(*F*^2^) is used only for calculating -*R*-factor-obs *etc*. and is not relevant to the choice of reflections for refinement. *R*-factors based on *F*^2^ are statistically about twice as large as those based on *F*, and *R*-factors based on ALL data will be even larger.

### Fractional atomic coordinates and isotropic or equivalent isotropic displacement parameters (Å^2^)

**Table:**

	*x*	*y*	*z*	*U*_iso_*/*U*_eq_	
Pb	0.60762 (4)	0.01636 (10)	0.85892 (3)	0.0213 (3)	
O1	0.6007 (6)	0.3400 (19)	0.5591 (4)	0.035 (2)	
O2	0.5381 (7)	0.120 (2)	0.6883 (5)	0.037 (3)	
O3	0.6905 (9)	−0.061 (2)	0.4106 (6)	0.032 (3)	
O4	0.8222 (6)	0.2863 (17)	0.4294 (4)	0.038 (2)	
N	0.7437 (6)	−0.1315 (19)	0.7170 (5)	0.024 (2)	
C1	0.7208 (12)	−0.0178 (18)	0.6321 (9)	0.021 (4)	
C2	0.7951 (11)	−0.058 (3)	0.5526 (9)	0.021 (3)	
C3	0.8932 (8)	−0.216 (2)	0.5715 (6)	0.027 (3)	
C4	0.9192 (9)	−0.325 (3)	0.6603 (7)	0.038 (4)	
C5	0.8383 (9)	−0.288 (3)	0.7300 (6)	0.036 (3)	
C6	0.6141 (8)	0.163 (3)	0.6231 (6)	0.023 (3)	
C7	0.7687 (9)	0.068 (2)	0.4580 (7)	0.019 (3)	
H3A	0.94410	−0.25180	0.52240	0.0320*	
H4A	0.98820	−0.41980	0.67230	0.0450*	
H5A	0.85060	−0.37490	0.78850	0.0430*	

### Atomic displacement parameters (Å^2^)

**Table:**

	*U*^11^	*U*^22^	*U*^33^	*U*^12^	*U*^13^	*U*^23^
Pb	0.0207 (5)	0.0242 (5)	0.0189 (5)	−0.0031 (1)	−0.0051 (3)	−0.0016 (1)
O1	0.035 (4)	0.045 (5)	0.026 (3)	0.015 (4)	0.007 (3)	0.012 (3)
O2	0.027 (4)	0.061 (6)	0.024 (3)	0.017 (5)	0.001 (3)	0.013 (4)
O3	0.039 (5)	0.031 (4)	0.027 (4)	0.006 (4)	−0.022 (4)	−0.013 (4)
O4	0.044 (4)	0.041 (5)	0.030 (3)	−0.007 (4)	−0.010 (3)	0.009 (3)
N	0.018 (4)	0.032 (5)	0.021 (3)	0.004 (3)	−0.003 (3)	0.004 (3)
C1	0.018 (7)	0.028 (6)	0.017 (6)	−0.002 (3)	0.000 (5)	−0.001 (3)
C2	0.006 (5)	0.037 (5)	0.021 (5)	0.002 (4)	−0.004 (4)	−0.010 (5)
C3	0.023 (5)	0.032 (6)	0.026 (4)	0.006 (4)	0.003 (4)	0.003 (4)
C4	0.029 (6)	0.054 (8)	0.031 (5)	0.018 (6)	−0.013 (4)	0.010 (5)
C5	0.041 (6)	0.046 (7)	0.021 (4)	0.009 (5)	−0.009 (4)	0.011 (4)
C6	0.018 (5)	0.031 (5)	0.021 (4)	0.006 (4)	−0.005 (3)	0.002 (4)
C7	0.008 (5)	0.030 (5)	0.019 (4)	0.006 (4)	−0.007 (4)	0.004 (4)

### Geometric parameters (Å, °)

**Table:**

Pb—N	2.651 (7)	N—C1	1.336 (14)
Pb—O1^i^	2.816 (7)	N—C5	1.327 (14)
Pb—O1^ii^	2.911 (6)	C1—C2	1.435 (18)
Pb—O2	2.592 (7)	C1—C6	1.499 (17)
Pb—O2^i^	2.566 (9)	C2—C3	1.379 (16)
Pb—O3^iii^	2.397 (9)	C2—C7	1.489 (16)
Pb—O3^ii^	2.754 (9)	C3—C4	1.385 (14)
Pb—O4^ii^	2.845 (7)	C4—C5	1.378 (14)
O1—C6	1.221 (13)	C3—H3A	0.9300
O2—C6	1.297 (12)	C4—H4A	0.9300
O3—C7	1.276 (14)	C5—H5A	0.9300
O4—C7	1.240 (12)		
			
O2—Pb—N	61.8 (2)	Pb^v^—O1—C6	150.1 (7)
O1^i^—Pb—O2	99.5 (2)	Pb^iv^—O1—Pb^v^	111.3 (2)
O2—Pb—O2^i^	71.1 (3)	Pb—O2—C6	118.5 (6)
O2—Pb—O3^iii^	124.6 (3)	Pb—O2—Pb^iv^	125.3 (3)
O1^ii^—Pb—O2	149.2 (2)	Pb^iv^—O2—C6	99.5 (7)
O2—Pb—O3^ii^	101.2 (3)	Pb^vi^—O3—C7	147.7 (8)
O2—Pb—O4^ii^	123.1 (2)	Pb^v^—O3—C7	88.8 (6)
O2—Pb—C7^ii^	121.1 (3)	Pb^vi^—O3—Pb^v^	123.4 (4)
O1^i^—Pb—N	139.5 (2)	Pb^v^—O4—C7	85.5 (6)
O2^i^—Pb—N	91.4 (2)	Pb—N—C1	117.7 (7)
O3^iii^—Pb—N	76.7 (3)	Pb—N—C5	122.1 (6)
O1^ii^—Pb—N	144.3 (2)	C1—N—C5	119.9 (9)
O3^ii^—Pb—N	102.6 (3)	N—C1—C2	122.4 (11)
O4^ii^—Pb—N	79.4 (2)	N—C1—C6	117.0 (10)
N—Pb—C7^ii^	97.8 (3)	C2—C1—C6	120.5 (10)
O1^i^—Pb—O2^i^	48.2 (2)	C1—C2—C3	114.7 (11)
O1^i^—Pb—O3^iii^	88.8 (3)	C1—C2—C7	122.2 (11)
O1^i^—Pb—O1^ii^	68.73 (19)	C3—C2—C7	123.1 (10)
O1^i^—Pb—O3^ii^	116.7 (3)	C2—C3—C4	123.0 (10)
O1^i^—Pb—O4^ii^	135.08 (17)	C3—C4—C5	117.2 (10)
O1^i^—Pb—C7^ii^	121.8 (2)	N—C5—C4	122.6 (9)
O2^i^—Pb—O3^iii^	75.1 (3)	O1—C6—O2	122.7 (10)
O1^ii^—Pb—O2^i^	113.1 (2)	O1—C6—C1	122.0 (9)
O2^i^—Pb—O3^ii^	158.7 (3)	O2—C6—C1	115.3 (10)
O2^i^—Pb—O4^ii^	153.8 (2)	O3—C7—O4	123.8 (9)
O2^i^—Pb—C7^ii^	167.3 (2)	O3—C7—C2	116.4 (9)
O1^ii^—Pb—O3^iii^	84.7 (3)	Pb^v^—C7—O3	66.1 (6)
O3^iii^—Pb—O3^ii^	123.4 (3)	O4—C7—C2	119.8 (9)
O3^iii^—Pb—O4^ii^	78.9 (3)	Pb^v^—C7—O4	70.3 (5)
O3^iii^—Pb—C7^ii^	98.4 (3)	Pb^v^—C7—C2	142.1 (7)
O1^ii^—Pb—O3^ii^	63.3 (2)	C2—C3—H3A	119.00
O1^ii^—Pb—O4^ii^	67.22 (18)	C4—C3—H3A	118.00
O1^ii^—Pb—C7^ii^	54.8 (2)	C3—C4—H4A	121.00
O3^ii^—Pb—O4^ii^	46.7 (3)	C5—C4—H4A	121.00
O3^ii^—Pb—C7^ii^	25.1 (3)	N—C5—H5A	119.00
O4^ii^—Pb—C7^ii^	24.2 (2)	C4—C5—H5A	119.00
Pb^iv^—O1—C6	89.5 (6)		
			
N—Pb—O2—C6	−27.3 (8)	O2—Pb—O4^ii^—C7^ii^	93.1 (6)
N—Pb—O2—Pb^iv^	−155.1 (5)	N—Pb—O4^ii^—C7^ii^	138.9 (6)
O1^i^—Pb—O2—C6	−169.0 (9)	O2—Pb—C7^ii^—O3^ii^	41.3 (7)
O1^i^—Pb—O2—Pb^iv^	63.2 (4)	O2—Pb—C7^ii^—O4^ii^	−102.4 (5)
O2^i^—Pb—O2—C6	−129.5 (9)	O2—Pb—C7^ii^—C2^ii^	144.2 (11)
O2^i^—Pb—O2—Pb^iv^	102.7 (4)	N—Pb—C7^ii^—O3^ii^	102.9 (6)
O3^iii^—Pb—O2—C6	−73.9 (10)	N—Pb—C7^ii^—O4^ii^	−40.7 (6)
O3^iii^—Pb—O2—Pb^iv^	158.3 (4)	N—Pb—C7^ii^—C2^ii^	−154.2 (12)
O1^ii^—Pb—O2—C6	126.9 (9)	Pb^v^—O1—C6—O2	−131.7 (11)
O1^ii^—Pb—O2—Pb^iv^	−0.9 (7)	Pb^iv^—O1—C6—C1	−175.5 (10)
O3^ii^—Pb—O2—C6	71.1 (9)	Pb^iv^—O1—C6—O2	3.8 (11)
O3^ii^—Pb—O2—Pb^iv^	−56.7 (4)	Pb^v^—O1—C6—C1	49.0 (19)
O4^ii^—Pb—O2—C6	25.9 (10)	Pb^iv^—O2—C6—C1	175.1 (8)
O4^ii^—Pb—O2—Pb^iv^	−101.9 (4)	Pb—O2—C6—C1	36.0 (13)
C7^ii^—Pb—O2—C6	54.5 (10)	Pb—O2—C6—O1	−143.4 (9)
C7^ii^—Pb—O2—Pb^iv^	−73.3 (5)	Pb^iv^—O2—C6—O1	−4.2 (12)
O2—Pb—N—C1	15.4 (7)	Pb^vi^—O3—C7—O4	136.7 (11)
O2—Pb—N—C5	−171.4 (9)	Pb^v^—O3—C7—O4	−42.2 (10)
O1^i^—Pb—N—C1	85.5 (8)	Pb^vi^—O3—C7—C2	−43.1 (19)
O1^i^—Pb—N—C5	−101.3 (8)	Pb^v^—O3—C7—C2	138.0 (9)
O2^i^—Pb—N—C1	83.0 (7)	Pb^vi^—O3—C7—Pb^v^	178.9 (15)
O2^i^—Pb—N—C5	−103.8 (9)	Pb^v^—O4—C7—O3	40.7 (10)
O3^iii^—Pb—N—C1	157.4 (8)	Pb^v^—O4—C7—C2	−139.5 (9)
O3^iii^—Pb—N—C5	−29.4 (9)	C5—N—C1—C2	0.1 (16)
O1^ii^—Pb—N—C1	−142.1 (7)	Pb—N—C1—C2	173.5 (8)
O1^ii^—Pb—N—C5	31.1 (10)	Pb—N—C1—C6	−5.1 (12)
O3^ii^—Pb—N—C1	−80.8 (7)	C1—N—C5—C4	4.1 (17)
O3^ii^—Pb—N—C5	92.4 (9)	C5—N—C1—C6	−178.5 (10)
O4^ii^—Pb—N—C1	−121.6 (7)	Pb—N—C5—C4	−169.0 (9)
O4^ii^—Pb—N—C5	51.6 (8)	C6—C1—C2—C3	176.8 (10)
C7^ii^—Pb—N—C1	−105.8 (7)	N—C1—C2—C3	−1.7 (16)
C7^ii^—Pb—N—C5	67.4 (9)	N—C1—C2—C7	179.7 (10)
O2—Pb—O1^i^—C6^i^	51.7 (7)	C6—C1—C2—C7	−1.9 (17)
O2—Pb—O1^i^—Pb^vii^	−150.3 (3)	N—C1—C6—O1	159.4 (10)
N—Pb—O1^i^—C6^i^	−5.4 (8)	N—C1—C6—O2	−20.0 (15)
N—Pb—O1^i^—Pb^vii^	152.5 (3)	C2—C1—C6—O1	−19.2 (17)
O2—Pb—O2^i^—Pb^i^	14.6 (4)	C2—C1—C6—O2	161.5 (10)
O2—Pb—O2^i^—C6^i^	−120.6 (7)	C1—C2—C3—C4	−0.7 (17)
N—Pb—O2^i^—Pb^i^	−44.8 (4)	C7—C2—C3—C4	177.9 (11)
N—Pb—O2^i^—C6^i^	179.9 (7)	C1—C2—C7—O3	−74.7 (15)
O2—Pb—O3^iii^—C7^iii^	136.3 (13)	C1—C2—C7—O4	105.5 (13)
N—Pb—O3^iii^—C7^iii^	95.1 (14)	C1—C2—C7—Pb^v^	10 (2)
O2—Pb—O1^ii^—Pb^vii^	72.2 (6)	C3—C2—C7—O3	106.7 (13)
O2—Pb—O1^ii^—C6^ii^	−59.0 (14)	C3—C2—C7—O4	−73.0 (15)
N—Pb—O1^ii^—Pb^vii^	−149.1 (3)	C3—C2—C7—Pb^v^	−168.9 (8)
N—Pb—O1^ii^—C6^ii^	79.8 (14)	C2—C3—C4—C5	4.5 (18)
O2—Pb—O3^ii^—C7^ii^	−144.8 (6)	C3—C4—C5—N	−6.3 (18)
N—Pb—O3^ii^—C7^ii^	−81.6 (6)		

Symmetry codes: (i) −*x*+1, *y*−1/2, −*z*+3/2; (ii) *x*, −*y*+1/2, *z*+1/2; (iii) *x*, −*y*−1/2, *z*+1/2; (iv) −*x*+1, *y*+1/2, −*z*+3/2; (v) *x*, −*y*+1/2, *z*−1/2; (vi) *x*, −*y*−1/2, *z*−1/2; (vii) −*x*+1, −*y*, −*z*+2.

### Hydrogen-bond geometry (Å, °)

**Table:**

*D*—H···*A*	*D*—H	H···*A*	*D*···*A*	*D*—H···*A*
C5—H5A···O3^iii^	0.93	2.57	3.164 (13)	122

Symmetry codes: (iii) *x*, −*y*−1/2, *z*+1/2.
